# Case report: Bladder preserving after maximal transurethral resection of the bladder tumor combined with chemotherapy and immunotherapy in recurrent muscle-invasive bladder cancer patients: A report of two cases

**DOI:** 10.3389/fmed.2022.949567

**Published:** 2022-08-01

**Authors:** Jiaquan Mao, Chunguang Yang, Sheng Xin, Kai Cui, Zheng Liu, Tao Wang, Zhiquan Hu, Shaogang Wang, Jihong Liu, Xiaodong Song, Wen Song

**Affiliations:** ^1^Department of Urology, Tongji Hospital, Tongji Medical College, Huazhong University of Science and Technology, Wuhan, China; ^2^Institute of Urology, Tongji Hospital, Tongji Medical College, Huazhong University of Science and Technology, Wuhan, China

**Keywords:** bladder-preserving therapy, programmed death-ligand 1 (PD-L1), immunotherapy, muscle-invasive bladder cancer (MIBC), case report

## Abstract

**Background:**

Cisplatin-based neoadjuvant chemotherapy combined with radical cystectomy (RC) plus pelvic lymph node dissection (PLND) is the preferred treatment option for muscle-invasive bladder cancer (MIBC). However, some patients are unable to tolerate RC or may have postoperative complications after RC. And most patients have a strong desire for bladder-preserving treatment. There are no reports on the efficacy of maximal transurethral resection of the bladder tumor (TURBT) in combination with chemotherapy plus tislelizumab for bladder-preserving in recurrent MIBC patients.

**Case presentation:**

We report two cases diagnosed with recurrent MIBC who achieved pathological complete response (pCR) and bladder-preserving after maximal TURBT combined with chemotherapy plus tislelizumab.

**Conclusion:**

Postoperative immunotherapy should be considered for all patients with recurrent MIBC who are eligible for immunotherapy. In addition, high programmed death ligand-1 (PD-L1) expression, high tumor mutation burden (TMB), and *TP53* mutation level can be combined to predict tislelizumab efficacy.

## Introduction

Bladder cancer is one of the top 10 most prevalent malignancies in terms of incidence worldwide (1). There are around more than 500,000 new cases of bladder cancer in the world and more than a third of these patients die from bladder cancer (2). Based on histomorphologic origin, bladder cancer can be categorized into urothelial carcinoma (UC), squamous cell carcinoma, adenocarcinoma, small cell carcinoma, and rhabdomyosarcoma, among others, the most common pathologic type is UC (≈80% of cases) (3). Approximately one-quarter of new cases are diagnosed as MIBC which is typified by susceptibility to recurrence, metastasis, and poor prognosis (4). According to the guidelines, neoadjuvant chemotherapy combined with RC plus PLND is the standard treatment that can prevent local recurrence and distant metastases of MIBC. However, some patients are unable to tolerate RC or may develop postoperative complications after RC, such as intestinal anastomotic leak, deep vein thrombosis, urinary tract infection, and deterioration of renal function (5). All of the above has a serious impact on the quality of life of the patients and therefore most patients wish to be treated with bladder-preserving. Consequently, it is extremely important to find bladder-preserving treatments that effectively control tumor progression. Trimodality bladder-preserving treatment (TMT) is a widely accepted and effective bladder-preserving treatment, but it has some drawbacks. Some patients after maximal TURBT experienced long-standing urinary and gastrointestinal toxicities during local radiotherapy (6). In addition, during local radiotherapy, the patient's bladder capacity is constantly changing due to changes in urine volume, making it very difficult to accurately locate the tumor for radiation therapy (6). Therefore, there is an ongoing need to find new and alternative bladder-preserving treatments to provide truly individualized treatment for different bladder cancer patients.

Immune checkpoint inhibitors have shown great potential in some solid tumors such as ovarian cancer, lung cancer, and melanoma (7–9). Tislelizumab is a novel humanized monoclonal antibody programmed death receptor-1 (PD-1) inhibitor (10). In the single-arm phase 2 trial (NCT04004221/CTR20170071), tislelizumab demonstrated clinical benefits in the treatment of multiple patients with metastatic UC and PD-L1 high expression who had failed platinum-based chemotherapy regimens, including tumor progression during neoadjuvant chemotherapy or within 12 months of adjuvant chemotherapy (10).

Here, we report two bladder-preserving treatments for recurrent MIBC with immunotherapy as an alternative to postoperative concurrent chemoradiotherapy, including neoadjuvant chemotherapy combined with maximal TURBT plus tislelizumab and maximal TURBT combined with adjuvant chemotherapy plus intravesical chemotherapy and tislelizumab.

## Case presentation

### Case 1

**Figure 1:**
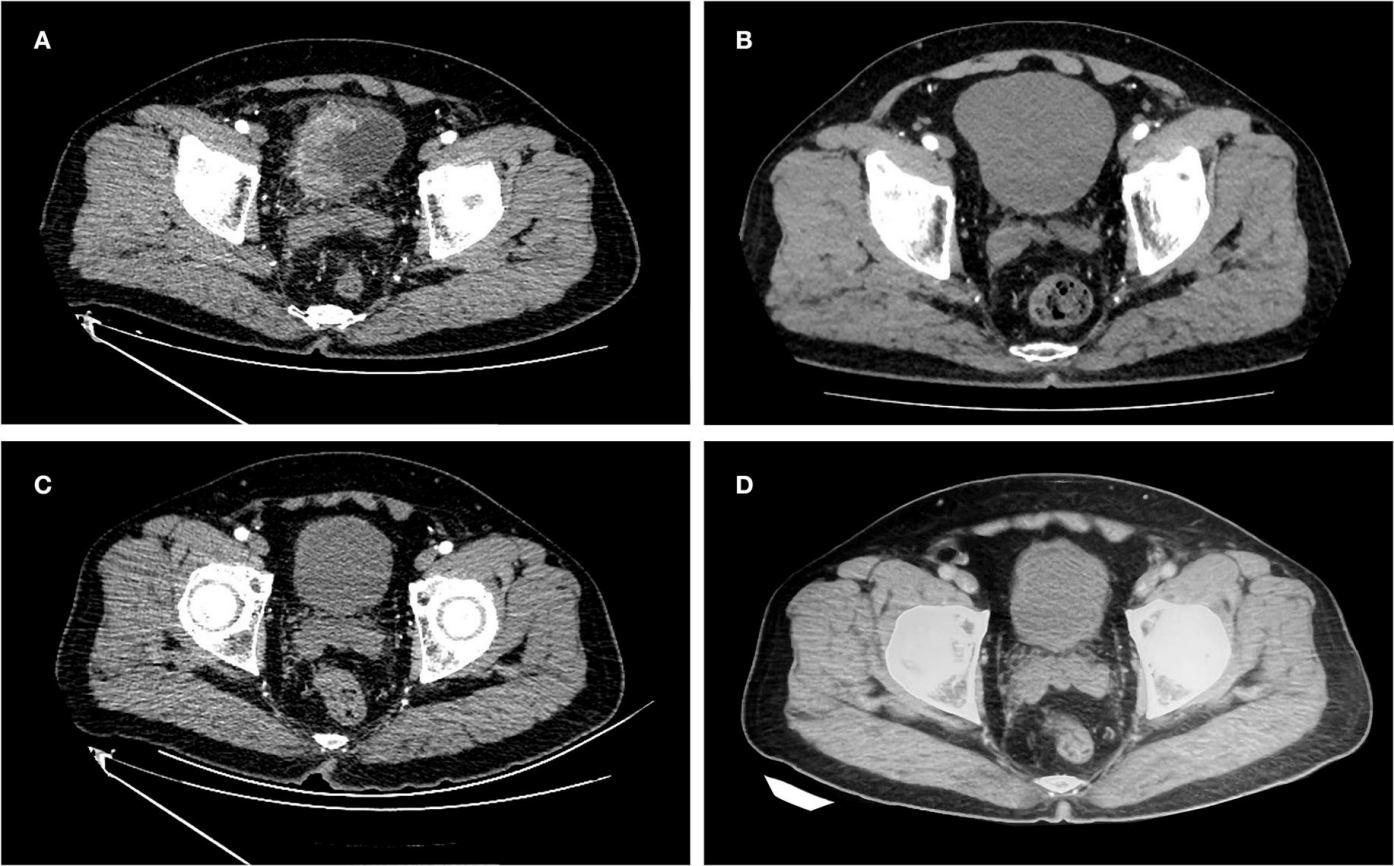
Images of the case 1 patient throughout the treatment. **(A)** Before treatment, abdomen enhanced CT showed localized invasion of the right wall lump of the bladder into the surrounding adipose tissue, invasion of the anterior bladder wall, and interruption of the continuity of the muscle layer. **(B)** After two courses of neoadjuvant chemotherapy, abdomen enhanced CT revealed a significantly reduced lump in the right wall of the bladder. **(C)** After maximal TURBT combined with tislelizumab, no tumor was seen on abdomen enhanced CT. **(D)** On February 18, 2022, the last follow-up abdomen enhanced CT showed no tumor recurrence.

A 61-year-old male was admitted to our institution on August 18, 2020, with the chief complaint of intermittent gross hematuria for more than 6 months. On May 20, 2020, the patient underwent TURBT at the local hospital, and the postoperative pathology indicated a high-grade UC of the bladder with muscle layer invasion. Because of the patient's strong desire to preserve the bladder, the patient underwent intravesical therapy with epirubicin. After 8 times of intravesical therapy, the patient again presented painless gross hematuria on July 31, 2020. The patient had a 3-year previous history of type 2 diabetes, with good glycemic control on oral metformin, and the rest of his personal history, family history, and physical examination in specialty was not exceptional. The patient's pelvic magnetic resonance imaging (MRI) and whole abdomen enhanced computed tomography (CT) on August 21 and 24, 2020 showed an irregular lump involving the right wall of the bladder with a maximum cross-sectional size of ≈59 × 30 mm, involving part of the anterior wall, with interrupted continuity of the muscular layer and localized invasion of the surrounding adipose tissue (Figure 1A). The patient was eventually diagnosed with cT_3b_N_0_M_0_ recurrent MIBC. We decided to give the patient neoadjuvant chemotherapy with a gemcitabine and cisplatin regimen. At the same time, the patient underwent biopsies by cystoscopy and genetic testing.

The purity of the collected tumor tissue samples of the patient was 50%, and the 520-gene panel was detected by next-generation sequencing (NGS) analysis. The TMB level was 35.89 mutations/Mb. We detected mutations in *ARID1A, BRCA2, TERT*, and *TP53* in the patient (Table 1). The combined positive score of the PD-L1 in the patient's tumor tissue was 70 and the tumor proportion score was 60% by immunohistochemistry (Supplementary Figures S1, S2).

**Table 1:** Results of genetic mutation testing of the patient tumor tissue.

**Gene**	**Position**	**Base alteration**	**Amino acid alteration**	**Mutation abundance**
*ARID1A*	Exon 3	c.1741C>T	p.Gln581*	15.78%
*BRCA2*	Exon 15	c.7516C>T	p.Gln2506*	10.79%
*TERT*	Promoter mutation	c.-65_-64delinsGA	N/A	10.42%
*TP53*	Exon 8	c.861G>C	p.Glu287Asp	19.25%
*TP53*	Exon 8	c.853G>A	p.Glu285Lys	18.49%

*It represents a mutation into a termination codon.

The patient started neoadjuvant chemotherapy on August 25, 2020, with a regimen of gemcitabine (1,000 mg/m^2^, days 1 and 8) and cisplatin (20 mg/m^2^, days 2, 3, and 4) in cycles of 21 days. In the third cycle of treatment, pelvic MRI and whole abdomen enhanced CT showed a slight local thickening of the right wall of the bladder, which was significantly smaller than before (Figure 1B). The patient had achieved a partial response in this cycle. On November 6, 2020, the patient completed all four cycles of neoadjuvant chemotherapy. The patient experienced second-degree toxic side effects including moderate urinary tract infection and lower abdominal pain during chemotherapy. After symptomatic treatments, the patient's symptoms were relieved.

On November 24, 2020, the patient underwent maximal TURBT (resection to the depth of the superficial muscle layer) and randomized biopsies by cystoscopy at our institution, and the postoperative pathology showed chronic inflammation of the bladder. The patient's pathological stage was downgraded to pT_0_.

Given the beneficial effects of anti-PD-1 therapy for progressive or locally advanced bladder cancer and the patient's pathological stage degradation results after neoadjuvant chemotherapy, the patient strongly requested immunotherapy and declined RC and radiotherapy. From December 2020 to the present, the patient has been treated with tislelizumab (200 mg, once per 21 days). To evaluate the efficacy of postoperative immunotherapy, the patient underwent a whole abdomen enhanced CT (Figure 1C) and biopsies by cystoscopy in March 2021 that showed normalcy and no tumor in the bladder. Thereafter, the patient underwent regular whole abdomen enhanced CT examinations at the local hospital all showing no tumor in the bladder and no sign of recurrence. During chemotherapy and immunotherapy, the patient underwent multiple chest CT and bone scintigraphy examinations with no abnormal findings. Until the end of follow-up on February 18, 2022, the patient maintained 15 months of no MIBC recurrence and a functional normal bladder, with no specific discomfort throughout the treatment course (Figure 1D).

### Case 2

**Figure 2:**
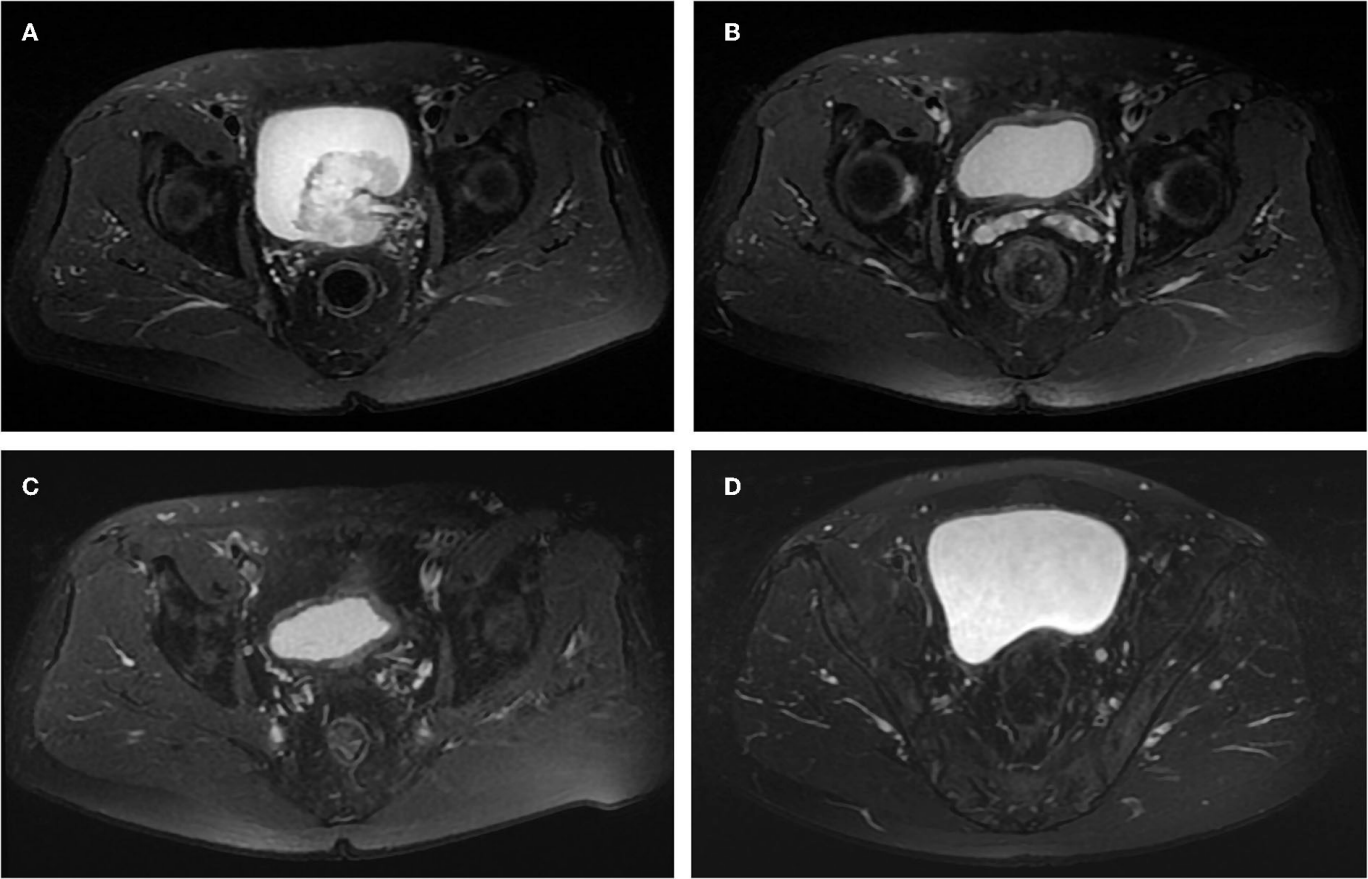
Images of the case 2 patient throughout the treatment. **(A)** Before treatment, pelvic MRI cross-sectional T_2_W showed a lump in the left posterior wall of the bladder and mild dilatation of the left ureter. **(B)** After the first maximal TURBT and four cycles of adjuvant chemotherapy plus tislelizumab, no tumor was seen on pelvic MRI cross-sectional T_2_W. **(C)** After the second maximal TURBT and gemcitabine intravesical therapy, no tumor was seen on pelvic MRI cross-sectional T_2_W. **(D)** On April 12, 2022, the last follow-up, no tumor was seen on pelvic MRI cross-sectional T_2_W.

A 67-year-old male was admitted to our institution on May 1, 2021, with the chief complaint of recurrent transient gross hematuria for more than half a month. The patient had no other specific signs and symptoms. The patient received TURBT 10 years ago, and the postoperative pathological evaluation indicated bladder papillary UC grade 2. Postoperatively, the patient received regular intravesical therapy with pirarubicin and cystoscopy, and no tumor recurrence was found. The rest of the patient's personal history, family history, and physical examination in specialty was not exceptional. This time, pelvic MRI and positron emission tomography/computed tomography revealed a large lump measuring ≈56 × 53 mm located in the left posterior wall of the bladder, with a mildly dilated left ureter (Figure 2A). The patient was eventually diagnosed with cT_2a_N_0_M_0_ recurrent MIBC. The patient expressed his desire to keep the bladder and refused genetic testing.

On May 8, 2021, the patient underwent the first diagnostic maximal TURBT (resection to the depth of the deep muscle layer). The postoperative pathological evaluation showed a high-grade UC of the bladder with muscle layer invasion. Some areas of tumor tissue showed classic, some areas with glandular differentiation and sarcomatoid changes.

Based on the patient's renal insufficiency, on May 18, 2021, the patient received adjuvant therapy with gemcitabine (1,000 mg/m^2^, day 1), carboplatin (60 mg/m^2^, day 2), and tislelizumab (200 mg, day3), in cycles of 21 days. On July 27, 2021, the patient completed all four cycles of adjuvant therapy. To evaluate the efficacy of adjuvant therapy, in August 2021, the patient underwent preoperative three urine cytologic examinations, fluorescence *in situ* hybridization examination, pelvic MRI (Figure 2B), whole abdomen enhanced CT, and the second diagnostic maximal TURBT, all of which showed normal, and no bladder tumor recurrence was observed. Postoperatively, the patient underwent two additional urine cytologic examinations that showed normal. We considered that the patient had obtained pCR during this period and the pathological stage was downgraded to pT_0_. Meanwhile, on August 23, 2021, the patient started postoperative intravesical therapy with a regimen of gemcitabine 1,000 mg once a week for a total of seven doses and then changed to once a month for a total of 10 doses. The patient developed second-degree toxic side effects including moderate urinary tract infection and skin damage during chemotherapy and immunotherapy. We treated the patient with anti-infective and anti-allergic symptomatic treatments and the patient recovered.

From September 2021 to March 2022, the patient received a regular pelvic MRI and maximal TURBT (resection to the depth of the serosal layer), all of which showed no signs of recurrence (Figure 2C). Throughout treatment, the patient underwent positron emission tomography/CT examination and no distant metastases of the tumor were detected.

Until the end of follow-up on April 12, 2022, the patient's pelvic MRI showed no significant abnormalities in the bladder (Figure 2D). The patient maintained a cancer-free status for 8 months.

## Discussion and conclusions

Since its widespread use in the treatment of malignant tumors, immunotherapy is effective in improving the survival of some tumor patients. However, some patients defined as PD-L1 positive have limited or no benefit after receiving immunotherapy (11). Tislelizumab, a novel humanized IgG4 monoclonal antibody (12), was approved by the National Medical Products Administration on April 10, 2020, for the treatment of locally advanced or metastatic combined PD-L1 high expression UC that had failed platinum-based chemotherapy including tumor progression during neoadjuvant chemotherapy or within 12 months of adjuvant chemotherapy (13). Previous studies have used ≥25% of tumor cell proportion score or immune cell proportion score and >1% of tumor-associated immune cells staining in the tumor area as cut-off selection to screen UC patients for treatment with tislelizumab (10, 14). Previously, TMT strategy was mostly applied for bladder-preserving treatment for MIBC patients. However, TMT has a negative impact on long-term normal bladder function, with ≈3% of patients experiencing reduced bladder capacity and 2% experiencing overactive bladder (15). Therefore, in the above cases, we replaced the postoperative concurrent chemoradiotherapy with tislelizumab until the end of follow-up without MIBC recurrence in both patients. The above results suggest that tislelizumab may be potentially beneficial in the treatment of recurrent MIBC patients.

Recent studies have found that TMB might predict the therapeutic efficacy of pembrolizumab in MIBC patients when the pretreatment TMB was ≥15 mutations/Mb (11, 16, 17). Based on the favorable treatment response to tislelizumab in the case 1 patient, we hypothesize that recurrent MIBC patients with high PD-L1 expression combined with high TMB (≥15 mutations/Mb) could benefit from tislelizumab.

The tumor suppressor gene, *TP53*, is located on chromosome 17p13. Exons 5–8 are human tumor mutation hotspots. A related small cohort study found that *TP53*-mutant tumor cells with adaptive immune resistance and hyperimmunogenic features improved patients' sensitivity to PD-1 inhibitors by promoting upregulation of PD-L1 expression and T-cell infiltration (18, 19). NGS results of the case 1 patient showed missense mutations in *TP53*(p.Glu287Asp, mutation level: 19.25%; p.Glu285Lys, mutation level: 18.49%), which might cause changes in the function of the encoded proteins and ultimately affected the patients' sensitivity to tislelizumab. Based on the excellent efficacy of postoperative immunotherapy in the patient, we hypothesize that the *TP53* mutation level could be used as a biomarker to predict the efficacy of immune checkpoint blockade therapy.

Our study suggests that maximal TURBT combined with chemotherapy plus tislelizumab may be an effective bladder-preserving treatment for recurrent MIBC patients. In addition, high PD-L1 expression, high TMB, and *TP53* mutation levels can be combined to predict immunotherapy efficacy.

## Data availability statement

The raw data supporting the conclusions of this article will be made available by the authors, without undue reservation.

## Ethics statement

The studies involving human participants were reviewed and approved by the Ethics Committee of Tongji Hospital, Huazhong University of Science and Technology. The patients/participants provided their written informed consent to participate in this study. Written informed consent was obtained from the individual(s) for the publication of any potentially identifiable images or data included in this article.

## Author contributions

JM and CY were responsible for writing the manuscript. SX and KC were responsible for data collection, data analysis, and data interpretation. ZL, TW, ZH, SW, and JL were responsible for image evaluation. XS and WS revised the manuscript. All authors read and approved the final manuscript.

## Conflict of interest

The authors declare that the research was conducted in the absence of any commercial or financial relationships that could be construed as a potential conflict of interest.

## Publisher's note

All claims expressed in this article are solely those of the authors and do not necessarily represent those of their affiliated organizations, or those of the publisher, the editors and the reviewers. Any product that may be evaluated in this article, or claim that may be made by its manufacturer, is not guaranteed or endorsed by the publisher.

## Abbreviations

RC: radical cystectomy
PLND: pelvic lymph node dissection
MIBC: muscle-invasive bladder cancer
TURBT: transurethral resection of the bladder tumor
pCR: pathological complete response
PD-L1: programmed death ligand-1
TMB: tumor mutation burden
UC: urothelial carcinoma
TMT: trimodality bladder-preserving treatment
PD-1: programmed death receptor-1
MRI: magnetic resonance imaging
CT: computed tomography
NGS: next-generation sequencing.
