# Supplementary figures and images for: Case report: Bladder preserving after maximal transurethral resection of the bladder tumor combined with chemotherapy and immunotherapy in recurrent muscle-invasive bladder cancer patients: A report of two cases
Source: Front Med (Lausanne). 2022 Aug 1;9:949567. doi: 10.3389/fmed.2022.949567 (PMC9377517; doi:10.3389/fmed.2022.949567)

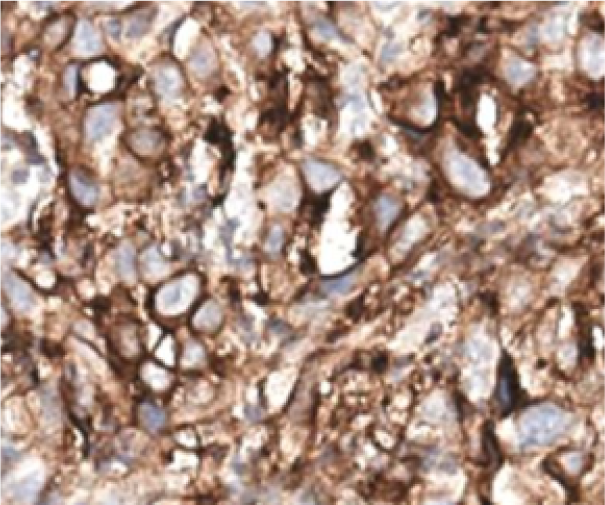

Supplement: Supplementary file 1 [file Image_1.TIF]

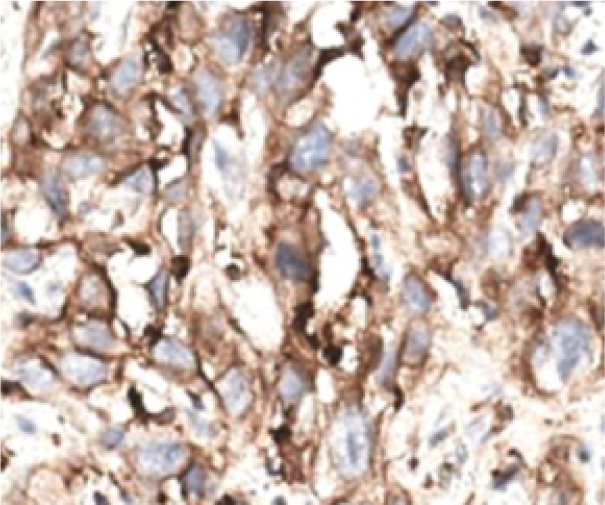

Supplement: Supplementary file 2 [file Image_2.TIF]
